# A double-edged sword: materiality classifications of sustainability topics

**DOI:** 10.1007/s11142-025-09908-1

**Published:** 2025-09-15

**Authors:** Max Göttsche, Paul A. Griffin, Florian Habermann, Frank Schiemann, Theresa Spandel

**Affiliations:** 1 Ingolstadt School of Management, Catholic University of Eichstätt-Ingolstadt, Ingolstadt, Germany; 2 Graduate School of Management, University of California Davis, Davis, CA USA; 3 Department of Accounting and Control, University of Lausanne, Lausanne, Switzerland; 4 Faculty of Social Sciences, Economics and Business Administration, University of Bamberg, Bamberg, Germany; 5 Faculty of Business, Economics and Social Sciences, University of Hamburg, Hamburg, Germany

**Keywords:** Corporate sustainability, Materiality classifications, Real effects, Sustainability incidents, G18, K22, L21, M14, M41

## Abstract

The Sustainability Accounting Standards Board (SASB) has classified sustainability topics as material or not material for investors. We leverage the staggered release of the SASB classifications from 2013 to 2016 to examine whether and how they prompt changes in U.S. firms’ sustainability performance. We measure sustainability performance using RepRisk scores, which reflect environmental, social, and governance (ESG) incidents. We find that RepRisk scores on sustainability topics classified as material decrease following the release of SASB classifications. Conversely, incident scores on nonmaterial sustainability topics increase. This suggests that firms improve their sustainability performance on topics the SASB deems relevant for investors while simultaneously performing worse on irrelevant topics. Firms adjust their internal sustainability policies to mirror these changes. The changes in sustainability performance occur primarily through two channels. We document that higher exposure to the classifications from shareholder pressure and sustainability-linked executive compensation prompts managers to prioritize sustainability topics classified as relevant for investors over irrelevant ones.

## Introduction

In light of growing concerns about the financial risks of adverse environmental and social outcomes, various stakeholders—including investors, regulators, and advocacy groups—have urged firms to adopt sustainable practices and increase transparency around them (Amel-Zadeh and Serafeim 2018; SEC 2024). Recognizing this need, the Sustainability Accounting Standards Board (SASB) began issuing industry-specific standards in 2013, including classifications that identify sustainability topics deemed financially material for investors (SASB 2022, 2023). As they provide a common understanding of the financial materiality of sustainability topics, the SASB’s classifications should, in theory, better align managers’ internal resource allocations with investors’ needs and preferences, leading to more efficient and positive sustainability outcomes (Friedman and Heinle 2016; Healy and Palepu 2001). However, in classifying certain sustainability topics as material, the SASB guidance implicitly identifies other topics as less or immaterial. What remains unknown from research is whether a standard setter’s distinction between material and immaterial sustainability topics has led firms to reallocate scarce resources away from the latter to improve their performance on the former—potentially neglecting sustainability outcomes that are relevant to stakeholders other than investors.

We investigate whether and how the release of the SASB’s materiality classifications changed managers’ resource allocation decisions and ultimately affected firms’ sustainability performance. We contend that managers could respond to the SASB’s materiality classifications *at the time of release* in one of three ways. One response is that managers are unsurprised by the classifications and investors’ views about them and make no reallocation. In this case, the sustainability performance of firms would not change after the release of the materiality classifications. A second response is that performance on material and immaterial topics would improve equally, reflecting increased or more efficient resource use with no reallocation. A third possibility is that by informing managers about investors’ preferences—which are crucial in shaping firms’ sustainability activities (Friedman and Heinle 2016; Hart and Zingales 2017)—the SASB classifications would prompt managers to reallocate resources, improving sustainability performance on material topics at the expense of weaker performance on immaterial topics.

Building on theoretical work on intrafirm resource allocation (Stein 1997) and evidence of trade-offs involving sustainability outcomes (Haffar and Searcy 2017), we hypothesize that this third possibility is the most likely. This view is further supported by studies on shareholder activism (Diaz-Rainey et al. 2024; Dimson et al. 2015) and sustainability-linked compensation (Bebchuk and Tallarita 2023; Cohen et al. 2023), which together illustrate two likely ways the SASB classifications feed back into firms, eventually enhancing managers’ awareness of material sustainability topics and changing their resource allocation decisions.

To test this hypothesis and identify firms’ potentially divergent shifts in sustainability performance, we implement a difference-in-differences design that leverages the staggered release of 77 SASB industry standards across 11 sectors from 2013 to 2016. This approach allows us to estimate the effect of the SASB’s materiality classifications on U.S. firms’ sustainability performance. Given recent advances in econometrics highlighting concerns of biased difference-in-differences estimates when treatment effects vary across units or over time (Baker et al. 2022; Callaway and Sant’Anna 2021; Goodman-Bacon 2021; Roth et al. 2023), we apply the Callaway and Sant’Anna (2021) estimator to account for potential dynamic and heterogeneous treatment effects. We use data from RepRisk (RepRisk 2023), Refinitiv (now LSEG), Institutional Shareholder Services (ISS) ESG, and Violation Tracker to measure firms’ changes in sustainability performance in response to the SASB standards.

Our study reveals two main findings. First, firms significantly reduced their material RepRisk scores (i.e., improved their material sustainability performance) after the release of the SASB standards. This suggests that the SASB classifications created a common understanding of financial materiality, aligning managerial decisions with investors’ needs. Conversely, we find a significant increase in immaterial RepRisk scores (i.e., deteriorated immaterial sustainability performance), suggesting that a managerial focus on material topics comes at the expense of immaterial ones. Notably, the SASB’s classifications are most influential in sectors where managers and investors previously lacked consensus on which sustainability topics were material. We conducted this test with SASB Industry Working Group disagreement data. Next, we address the concern that RepRisk’s reliance on third-party sources, particularly media coverage, may result in the systematic underrepresentation of incidents that receive limited public attention (Johnson 2020). To mitigate this potential measurement bias, we complement our main analyses with alternative sustainability performance measures, namely, workplace safety and health violations recorded by Violation Tracker and firms’ carbon emission intensity based on ISS data. The results based on these alternative measures support our main findings, alleviating concerns that media selection of incidents to cover may bias our main results.

To test whether the observed changes in material and immaterial sustainability performance are driven by shifts in managerial focus, we examine changes in firms’ internal sustainability policies. We conduct cross-sectional tests and use Refinitiv data on firms’ internal sustainability policies (Refinitiv 2023) as proxies for managers’ reallocation decisions. These findings reveal an increase in material sustainability policies and a decrease in immaterial policies following the release of the SASB standards. This suggests that managers prioritized sustainability topics deemed material by the SASB standards over immaterial ones. Moreover, the observed timing between internal policy changes and subsequent shifts in sustainability performance is consistent with a shift in managerial attention following the standard’s release. Specifically, we find that managers reallocated resources from immaterial to material policies immediately after the standard’s release, which subsequently—within 10 to 12 months—initiated an improvement in material sustainability performance and a decline in immaterial performance, with these effects increasing over time.

Our hypothesis is based on the idea that the release of the SASB’s materiality classifications guided managerial decision-making. Moreover, altered investor preferences, which fed back into firms through shareholder activism and sustainability-linked compensation, may have amplified the alignment of managerial decisions with SASB’s materiality classifications. Consequently, we examine these two channels as explanations of how investor preferences influence firms. We first build on evidence that shareholder activism enhances managers’ awareness of sustainability issues (Cunat et al. 2012; Diaz-Rainey et al. 2024; Dimson et al. 2015; Flammer et al. 2021). We contend that managers facing at least one sustainability-related shareholder proposal before the release of the SASB standards would emphasize aligning their sustainability performance with the SASB’s materiality classifications. Based on FactSet shareholder proposal data (FactSet 2023), we find that pre-SASB shareholder pressure amplifies firms’ focus on material sustainability topics in the years following the standards’ releases.

Second, while research offers inconclusive evidence on the impact of sustainability-linked compensation on sustainability performance (Bebchuk and Tallarita 2023; Cohen et al. 2023), we argue that its effects would manifest when distinguishing between material and immaterial sustainability performance. Consistent with this argument, we find that sustainability-linked compensation plans introduced after the release of the SASB standards shift managers’ focus toward material sustainability topics, with no significant change in attention to immaterial topics. Focusing on the compensation plans of the largest firms in our sample, we also find support for the Bebchuk and Tallarita (2023, p. 37) claim that “the use of these [sustainability-linked compensation] metrics could well ultimately hurt, not serve, aggregate stakeholder welfare.” In sum, these additional analyses provide strong evidence that shareholder pressure and sustainability-related compensation help induce shifts in sustainability performance.

Our study makes three main contributions. We first extend research on the effects of the SASB standards by examining market-wide real effects following the release of the standards. We thereby extend the work of Bochkay et al. (2025), who show that the SASB standards are a coordination device for sustainability information, influencing both managerial and investor focus on sustainability topics in earnings calls. We further complement Bochkay et al. (2022) by demonstrating trade-offs in firms’ material and immaterial sustainability performance and resource allocations, following the SASB standard releases, independent of firms’ later adoption of the standards.

Second, we contribute to the literature on the real effects of sustainability standards by introducing the dimension of materiality classifications. While most research focuses on firm disclosures following the adoption of sustainability disclosure standards (Bonetti et al. 2024; Christensen et al. 2017, 2021; Fiechter et al. 2022), we show that real effects can occur as early as the release of the standards. By contrast to studies that show reduced greenhouse gas emissions (Jouvenot and Krueger 2021) and improved water quality (Bonetti et al. 2024) in response to firms’ disclosures after adopting new standards, we demonstrate that sustainability standards introducing materiality classifications drive firms’ resource allocation decisions immediately and before firms’ adoption.

In addition, we show for the first time that standards that generate a common understanding of the financial materiality of sustainability topics have an offsetting downside: improvements in material sustainability topics come at the expense of degraded performance on sustainability topics classified as immaterial. This represents an important insight because studies mainly investigate trade-offs between firms’ outcomes on sustainability topics, such as emissions or mine safety, and financial performance outcomes, such as productivity or profitability (Christensen et al. 2017; Downar et al. 2021). Our study, by contrast, reveals that firms’ responses to market-wide materiality classifications represent a trade-off between material and immaterial sustainability topics. Taken together, our findings reveal that a standard setter’s materiality classifications *at the time of their initial release* shifted firms’ attention and resources toward the interests of the targeted stakeholder group (i.e., investors). However, this guidance also forged a double-edged sword as resources were diverted away from topics deemed immaterial, leaving stakeholders affected by neglected topics to bear the consequences.

## Institutional background, related literature, and hypotheses

### SASB, standard setting, and related literature

The SASB was founded in 2011 to set standards to guide firms in “disclosing financially material sustainability information for investors.” It subsequently merged with the Value Reporting Foundation and later the IFRS Foundation, the overseeing body of the International Sustainability Standards Board (ISSB). Presently, firms subject to the ISSB standards shall consider the materiality map of the SASB as guidance on their disclosure of sustainability risks, opportunities, and outcomes.^1^ The core goal of the mapping exercise, according to SASB, is to identify on an industry level which sustainability topics are relevant to investors and thus material regarding financial performance and firm value.^2^

To accomplish this goal, the SASB undertook a two-phase standard-setting process. With Phase I, 11 provisional standards were sequentially established and released between July 2013 and March 2016. In Phase II, these provisional standards underwent codification, and all Codified Standards were collectively released in November 2018. The staggered release of the 11 provisional standards established pivotal moments for each sector and its associated industries: they marked the initial dissemination of significant information and guidance regarding materiality assessments for investors, focusing on implementation rather than merely soliciting feedback. For this reason, these 11 release dates serve as the event dates in our empirical analyses (Appendix A).

Initially, the SASB’s research team conducted an internal research phase to gather evidence pertinent to specific industries, particularly concerning the relevant material sustainability disclosure topics and corresponding accounting metrics. The key findings from this research phase were synthesized into industry briefs and surveys, which were subsequently disseminated to SASB Industry Working Groups during the external development phase. Comprising representatives from three distinct stakeholder groups—firms, investors, and public interest groups—the working groups were established through an open enrollment process followed by active outreach.^3^ The SASB, however, mostly included capital-market-oriented stakeholders across the industry groups, ensuring that the standards aligned with the interests of investors over other stakeholders.

**Fig. 1:**
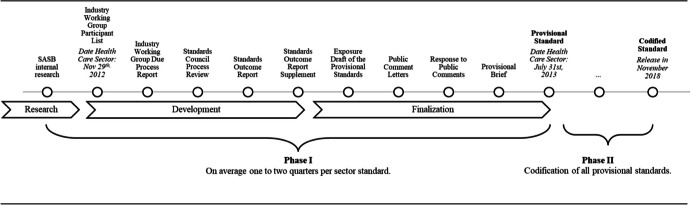
**Timeline for the SASB standard-setting process**. The figure shows the detail of Phase I of the standard-setting process. In addition, the figure shows the dates for the first and last steps of Phase II for the healthcare sector

In the next step, working group participants were asked to review the industry briefs and complete surveys, including the tasks of eliminating, adding, and prioritizing sustainability disclosure topics from a list of high-priority material topics. The SASB then assimilated the responses to write an exposure draft, which served as a preliminary version of provisional standards. After the finalization phase, which included an additional public consultation, the provisional standards were released for use and implementation between 2013 and 2016. This entailed additional contextual information, such as the standards’ objectives, target users, and scope, along with the “Material Sustainability Topics and Accounting Metrics” pertinent to each industry within a given sector. The SASB also published a visualization by illustrating the material disclosure items encompassed in the standards with a “materiality map” accompanying each standard.^4^ Figure 1 provides an overview of the key subphases of the Phase I dates for the first sector (healthcare) at both the inception and ending stages. As the initial publication of the materiality map for the healthcare sector occurred on July 31, 2013, this month serves as our first event date.

Considering the standard-setting process, the brief interval between SASB Industry Working Groups’ access to the SASB’s initial materiality classification (during the external development phase) and the subsequent release of provisional standards should mitigate concerns about firms’ anticipation effects. In addition, because “the timing of the release of SASB standards across industries was pre-determined in 2011 … and independent of companies’ existing disclosure policies [or performance]” (Grewal et al. 2021, p. 530), another concern about identification—that is, an emphasis on standard setting for industries that are more sensitive to sustainability topics—is also alleviated.

While the literature on SASB standards is recent, some studies suggest that the standards function as a credible classification framework of material sustainability topics for investors (Grewal et al. 2021; Matsumura et al. 2022). For example, Bochkay et al. (2022) show that the number of negative sustainability news items declines for firms after adopting a SASB standard in their sustainability disclosures. Focusing on investors’ use of the standards, other studies show that the SASB standards function as a coordination device in earnings calls (Bochkay et al. 2025) or a prioritization framework for investors, who adjust their weightings on sustainability topics after the standard releases (Spandel et al. 2022). Beyond empirical insights on SASB standards’ acceptance and usage, the SASB standards have been widely applied in business practice (Bradley 2023; SASB 2018, 2023). In addition, the ISSB’s disclosure standards rely on the SASB materiality framework (IFRS 2023).

### Hypothesis development

Information on firms’ sustainability performance can provide monitoring benefits for investors, helping them uncover insufficient performance and pressure managers to improve sustainability performance. Managers may then respond by improving firm performance (Christensen et al. 2021; Healy and Palepu 2001). In settings where mandatory disclosure regulations are introduced, recent work corroborates the role of pressure by linking firm disclosure and subsequent stakeholder pressure to reductions in emissions (Jouvenot and Krueger 2021) and improvements in water quality (Bonetti et al. 2024).

By contrast, the SASB’s materiality classifications represent a shock to the information environment of investors and managers, offering benefits as early as the time of the standard releases and irrespective of firms’ subsequent adoption of the standards. In other words, the SASB standards likely establish a common understanding of financial materiality for sustainability topics, thus informing managers which topics matter most to investors. For example, BlackRock CEO Larry Fink states: “SASB provides a clear set of standards for reporting sustainability information” (Fink 2020). Consequently, managers are not immune to the SASB’s choice of topics considered to be material within their respective industry, partly because, during standard-setting, investor representatives as members of the SASB Industry Working Groups have agreed that these topics are the most value-relevant (Krueger et al. 2020). This aligns with managers’ fiduciary duty to consider the value relevance of (material sustainability) topics to create shareholder value (Welch and Yoon 2023).

Studies demonstrate that not only new information but also already publicly available information when disclosed accessibly can affect firms’ sustainability performance. While most evidence stems from mandatory disclosure settings (Christensen et al. 2017; Downar et al. 2021), we argue a SASB standard release operates similarly. Although the materiality of sustainability topics can be defined by investors before SASB’s materiality classifications are released, the outcome of the materiality assessment (i.e., which topics are material) can differ among investors.^5^ Similarly, firms’ materiality assessments of which sustainability topics to manage and disclose can differ even within the same industry because of firms’ different goals (Christensen et al. 2021). This should change, however, after the SASB provides the first market-wide set of industry-specific materiality classifications via its standards.

Consequently, after each standard release, a common understanding of materiality in sustainability topics is likely to emerge, which can reduce data-gathering and decision-making costs for investors (Blankespoor et al. 2020). The statement of Larry Fink: “We are asking the companies that we invest in … to publish a disclosure in line with industry-specific SASB guidelines” (Fink 2020) implies that BlackRock expects benefits through SASB-aligned disclosures. At the time of a standard release, managers may anticipate that this new materiality framework will align investors’ preferences with SASB’s materiality classifications and guide future investor decision-making. Shareholder proposals on sustainability topics deemed material by SASB may also increase in frequency and gain greater support or more attention from managers and corporate bodies (e.g., governance and sustainability, nominations, or remuneration committees), eventually influencing managerial compensation and career trajectories. For example, Bochkay et al. (2025) show that both analysts and managers increasingly discuss and focus on material sustainability topics in earnings calls following the release of the SASB standards. Taken together, the common understanding of materiality tends to drive managers’ prioritization of material sustainability topics.

In addition, the industry-specific nature of the SASB’s materiality classifications may further align managers’ resource allocation decisions with the sustainability activities of their peers (Cao et al. 2019; Tomar 2023). Following the release of SASB’s materiality classifications, managers in their respective industry know whether their focus on sustainability topics is consistent with and comparable among peers in their industry. Managers are also aware that their firm would not be competitively disadvantaged through compliance or peers’ knowledge of their progress (e.g., operating and investment activities) to improve performance on material sustainability topics. In their study of firms’ adoption of SASB standards, Bochkay et al. (2022) find that firms are likelier to adopt SASB standards after peer adoption.

In summary, managers can be expected to respond to or anticipate shareholder pressure and allocate resources toward material sustainability topics following the release of SASB’s materiality classifications. Therefore, we state our first hypothesis as follows:

- *H1.* Firms’ sustainability performance on material topics improves after the release of the SASB standards.

The effect of the SASB’s materiality classifications on firms’ performance comprising sustainability topics classified as immaterial for investors is more ambiguous. This is because, to our knowledge, no theory addresses firms’ resource allocation decisions that may trade-off material and immaterial sustainability performance.^6^

Nevertheless, we can draw on theoretical work on intrafirm resource allocation (Stein 1997) and studies on the trade-offs between financial and sustainability performance (Haffar and Searcy 2017). The former study shows the importance of headquarters in making strategic investment decisions. In our case, we argue that following the release of the SASB standards, top management—anticipating shareholder pressure on material topics in the future—is more likely to make material sustainability topics their strategic priority. Bochkay et al. (2025) document an increased managerial focus on material topics in earnings calls following the release of the SASB standards, supporting the idea that managers also internally directed their strategic focus on material topics leading to new or more ambitious targets for these material topics.

These targets, in turn, lead to cost–benefit trade-offs tied to a “measurement-management” tension when resources are limited within firms, which justifies “allocating resources to measured targets over others” (Haffar and Searcy (2017, p. 504). Because of this tension, managers allocate resources to material topics due to their value relevance while deprioritizing immaterial ones. An example of resource reallocation following a SASB standard release is when firms hire experts on material sustainability topics or retrain employees previously specialized in immaterial topics, redirecting their efforts to achieve the new or more ambitious targets for material topics set by the top management. Such a shift in resource allocation also aligns with managers’ fiduciary duty to allocate resources efficiently to maximize firm value.

Another possibility, though, is that managers are unsurprised (i.e., they already agree with investors’ views on materiality) and do not respond to the SASB standard releases. Building on our arguments regarding intrafirm resource allocation and the measurement-management tension, we state our second hypothesis as an adverse effect on immaterial sustainability performance against the null hypothesis of no reallocation—that is, no effect on performance on immaterial topics—as follows:

- *H2*. Firms’ sustainability performance on immaterial topics declines after the release of the SASB standards.

## Sample and data

### Sample

Our initial sample contains 1,691,475 Daily RepRisk incident observations, including 73,950 ESG incidents concerning 797 large public U.S. firms from 2007 to 2020. We restrict our investigation of the potential real effects of SASB standards to a sample of U.S.-listed firms because the SASB is a U.S.-based organization that targets U.S.-listed firms and investors with its standards. Beyond that, the U.S. context is particularly suitable for studying the effects of standards, as no other market-wide sustainability disclosure standards were introduced in the United States during the sample period. By contrast, the implementation of the Non-Financial Reporting Directive by the European Union in 2014 (EU 2014), which took effect in 2017, could impact European firms’ sustainability performance, distorting any effect of the SASB standard releases in Europe.

Our main findings (Sect. 5.1) are based on monthly RepRisk incident data starting in January 2013 (six months before the first SASB standard release in the healthcare sector) and ending in February 2016 (the month before the last standard release for the infrastructure sector). We then match the incident data with accounting data (e.g., total assets and sales growth). After removing observations with missing values, the dataset includes 24,408 firm-month observations. Panels A and B of Table 1 show the sample distribution by year and sector. Panel A shows that our observations are distributed evenly by year. Panel B illustrates that the observations differ by sector, with a relatively low number of firms in the renewable resources sector. Our tests incorporate whether this unequal distribution of observations across industries could influence our results (Goodman-Bacon 2021). Panel C of Table 1 provides summary statistics for the monthly data. Within the range of 0 to 100, the mean material RepRisk score (*matRRESG*) is 20.90, whereas the mean immaterial RepRisk score (*immatRRESG*) = 11.44. The firms studied are mostly profitable (mean *ROA* = 5.16%) and owned by institutional investors (mean *Inst. Ownership* = 79.34%).

**Table 1:** Sample description and summary statistics

**Panel A**. Sample distribution by year (January 2013 to February 2016)											
		2013	2014		2015		2016		Total		
No. of observations		5,854	6,130		6,194		6,230		24,408		
**Panel B**. Sample distribution by sector											
Sector			No. of observations				Percentage				
Consumer Goods			2,849				11.67				
Extracting and Minerals Processing			2,683				10.99				
Food and Beverage			2,833				11.61				
Financials			2,596				10.64				
Healthcare			2,165				8.87				
Infrastructure			3,182				13.04				
Renewable Resources			144				0.59				
Resource Transformation			2,652				10.87				
Services			1,323				5.42				
Technology			2,776				11.37				
Transportation			1,205				4.93				
Total			24,408				100.00				
**Panel C**. Summary statistics main analysis: monthly data (January 2013 to February 2016)											
Statistic	No. obs		Mean	St. Dev		Min	Pctl (25)	Pctl (75)			Max
RepRisk scores											
*matRRESG*	24,408		20.90	17.64		0.00	0.35	34.17			73.41
*immatRRESG*	24,408		11.44	11.65		0.00	1.45	19.06			59.52
Refinitiv accounting data											
*Total Assets $US 100,000*	24,408		57,713	203,717		103	4,963	36,188			2,572,274
*ROA (%)*	24,408		5.16	8.17		−85.47	1.52	8.45			137.22
*Sales Growth (%)*	24,408		4.51	24.85		−85.32	−3.42	9.67			387.23
*Inst. Ownership (%)*	24,408		79.34	19.90		0.00	79.34	91.75			100.00
**Panel D**. Summary statistics additional analysis: yearly data (2011 to 2020)											
Statistic	No. obs		Mean	St. Dev		Min	Pctl (25)	Pctl (75)		Max	
RepRisk scores											
*matRRESG*	5,028		21.00	17.39		0.00	0.62	33.52		74.91	
*immatRRESG*	5,028		11.45	11.68		0.00	1.48	18.97		58.79	
Refinitiv corporate sustainability policy scores											
*materialESGpol*	5,028		40.47	25.02		0.00	20.54	61.52		84.40	
*immaterialESGpol*	5,028		39.39	23.63		0.00	23.08	58.24		85.32	
Refinitiv accounting data											
*Total Assets (in $US 100,000)*	5,028		61,112	214,449		78.00	5,168	38,575		3,386,071	
*ROA (%)*	5,028		5.03	8.17		−85.47	1.52	8.45		137.22	
*Sales Growth (%)*	5,028		9.86	319.27		−85.32	−2.45	10.49		22,587	
*Inst. Ownership (%)*	5,028		78.97	20.27		0.00	71.10	91.81		100.00	
*Additional data*											
*IntensityScope1 (ISS)*	4,403		327.53	1061.13		0	2.48	64.93		19,192.86	
*IntensityScope2 (ISS)*	4,403		60.08	181.54		0	0.17	410.69		6,738.25	
*FineSafetyVio (Violation Tracker)*	5,028		1.79	12.34		0	0	0.35		344.88	
*Number of ESG proposals (Factset)*	2,382		0.78	1.37		0	0	1		18	
*preSASBprop*	2,297		0.10	-		0	0	0		1	
*CompInitiator (Refinitiv)*	4,532		0.43	-		0	0	1		1	
*ESGCompS&P100 (Bebchuk and Tallarita compensation data)*	433		0.53	-		0	0	1		1	

Note that RepRisk scores range between 0 (best performance) and 100 (worst performance). Refinitiv scores range between 0 (worst performance) and 100 (best performance).

Panel D of Table 1 shows summary statistics for the full sample, containing yearly observations from 2011 to 2020. The sample comprises 5,028 firm-year observations (Sect. 5.2) and is reduced as described in the respective analysis Sects. (5.3 and 6.1 to 6.3). We further access Scope 1 and 2 emissions from ISS (ISS 2023) and measures of fines for workplace safety and health violations from Violation Tracker (Violation Tracker 2023) to mitigate concerns about measurement bias in (*im*)*matRRESG*. We use sustainability policy scores from Refinitiv (now LSEG; 2024) to proxy for resource allocation and FactSet shareholder proposal details to proxy for shareholder pressure. We use sustainability compensation details from Refinitiv and Bebchuk and Tallarita (2023) to test whether sustainability-linked compensation affects subsequent sustainability performance. Panel D of Table 1 provides summary statistics for these variables. Appendix B describes the variables

### Data

We use incident data from RepRisk as our measure of firms’ sustainability performance. RepRisk is a Swiss-based data provider that employs a rules-based methodology, focusing exclusively on third-party information, such as print and online media, newsletters, and governmental reports in 23 languages. This approach ensures independence from firms’ self-reported data, which may be greenwashed. However, the reliance of RepRisk on third-party information, particularly media coverage, introduces a potential limitation. For example, media coverage might systematically ignore smaller incidents, as these might not be seen as relevant for news articles. Indeed, Johnson (2020) highlights that the Occupational Safety and Health Administration’s press release policy only publicizes workplace safety violations that exceed a fine cutoff. This approach leaves smaller violations unreported, even though they represent low performance, thus introducing measurement bias. To address this concern, we measure sustainability performance with alternative data: workplace safety or health violations from Violation Tracker and carbon emission intensity from ISS ESG. These measures allow us to corroborate our findings and mitigate the concern of measurement bias.

**Fig. 2:**
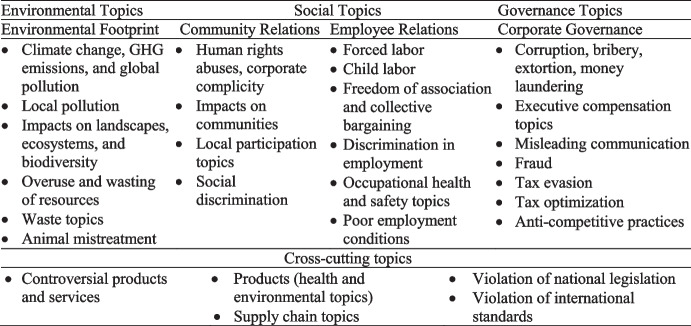
**RepRisk ESG framework**

Despite its potential limitation, RepRisk offers two advantages that suit it for this study. First, RepRisk’s evaluation methodology is consistent over the sample period (2011–2020). Second, and most importantly, RepRisk data allow us to derive RepRisk ESG scores (denoted *RRESG*) based on the SASB materiality classifications at the industry level.^7^ Specifically, we construct scores for material sustainability incidents (denoted *matRRESG*) by aligning industry-level RepRisk data with SASB classifications and calculate scores for immaterial incidents (*immatRRESG*) by subtracting *matRRESG* from *RRESG*.^8^ Figure 2 below illustrates that *RRESG* spans 28 sustainability topics, encompassing 23 topics distributed across the three ESG pillars and five cross-cutting topics linked to more than one pillar.

The application of RepRisk data in research settings (e.g., Bansal et al. 2022; Dai et al. 2021; She 2022) and in practice supports the validity of the data. For example, She (2022) uses RepRisk to measure human rights abuses; and Li and Wu (2020) use RepRisk to study firms’ sustainability performance following United Nations Global Compact (UNGC) engagements. Stock market analysts (Luo et al. 2015) and firms (RepRisk 2022) use RepRisk to make investment and supplier-contracting decisions.

We illustrate the RepRisk ESG framework in the context of this study with the example of American Airlines Group. As of the end of fiscal year 2018, the firm’s overall RepRisk score (*RRESG*) is 41.51. As American Airlines Group operates in the airline industry, incidents are classified as material under SASB standards when RepRisk analysts associate them with greenhouse gas emissions, labor practices, critical incident risk management, or competitive behavior. The score including material sustainability incidents (*matRRESG*) was 29.72. All remaining incidents, spanning topics, such as customer privacy or water and wastewater management, are classified as immaterial and contribute to the immaterial RepRisk score (*immatRRESG*), which is 11.79 (derived by subtracting 29.72 from 41.51).

## Real effects of materiality classifications

We contend that managers act following the release of the SASB standards because the standards create a common understanding between investors and managers about what sustainability topics within an industry are material. Thus, we can exploit the standard release months as shocks to management decision-making. Appendix A lists the 11 sector release dates, from July 2013 (healthcare sector) to March 2016 (infrastructure sector). However, despite the standard-setting process providing precise treatment months, two identification concerns remain. One occurs because the standards were released across all industries within each of the 11 sectors, resulting in the absence of a never-treated control group. Beyond that, the conventional way to address the economic impact of staggered shocks in difference-in-differences designs can generate a bad comparisons problem (Callaway and Sant’Anna 2021; Goodman-Bacon 2021; Roth et al. 2023), leading to biased inferences (Baker et al. 2022).

We address these identification concerns and generate unbiased inferences following Callaway and Sant’Anna (2021).^9^ In particular, their difference-in-differences estimator allows for heterogeneity in the average treatment effect for the treated units (*ATT*) across groups (i.e., sectors) and over time. Using their nomenclature, we define the average treatment effect for units that are members of cohort group *g* at time *t*, which is a unit cohort-time-specific treatment effect, as follows:

1 $$ATT\left(g,t\right)= {\mathbb{E}}\left[{Y}_{t}\left(g\right)-{Y}_{t}\left(0\right) \right| {G}_{g}=1].$$

Equation (1) refers to sector cohort $g$ at time $t$. ${G}_{g}$ is defined as a binary variable where ${G}_{g}$ is one if a firm is treated at time $t$. The dependent variable *Y*_*t*_* (g)* for each firm in group *g* is *matRRESG* or *immatRRESG*. The variable *Y*_*t*_ (0) represents a not-yet-treated unit’s potential outcome at *t*. Not-yet-treated units serve as controls until they eventually become treated. In our sample, firms in the infrastructure sector serve as never-treated control units, as the SASB released its standards for industries in this sector last. Following Callaway and Sant’Anna (2021), we estimate $ATT\left(g,t\right)$ using their doubly robust procedure. We further use firm-level clustered bootstrapped-based standard errors.

Because we are interested in an aggregated causal parameter to test our hypotheses, we estimate the cumulative *ATT* across all sectors $\theta$ at different treatment exposure lengths $e$, defined as $\theta \left(e\right)$ (Callaway and Sant’Anna 2021, pp. 208, 209). By averaging $ATT\left(g,t\right)$ over all *g* for a respective exposure length *e*, we can observe *ATT* $\theta \left(e\right)$. Thus, with *ATT* $\theta \left(e\right)$, we can understand the dynamic effects of the SASB standard releases on firms’ material and immaterial sustainability performance. As a final step, we can average *ATT* $\theta \left(e\right)$ across all exposure lengths $e$ to observe the overall treatment effect ${ATT \theta }^{Overall}$.

## Results

### Baseline results: Treatment effects by exposure length

**Fig. 3:**
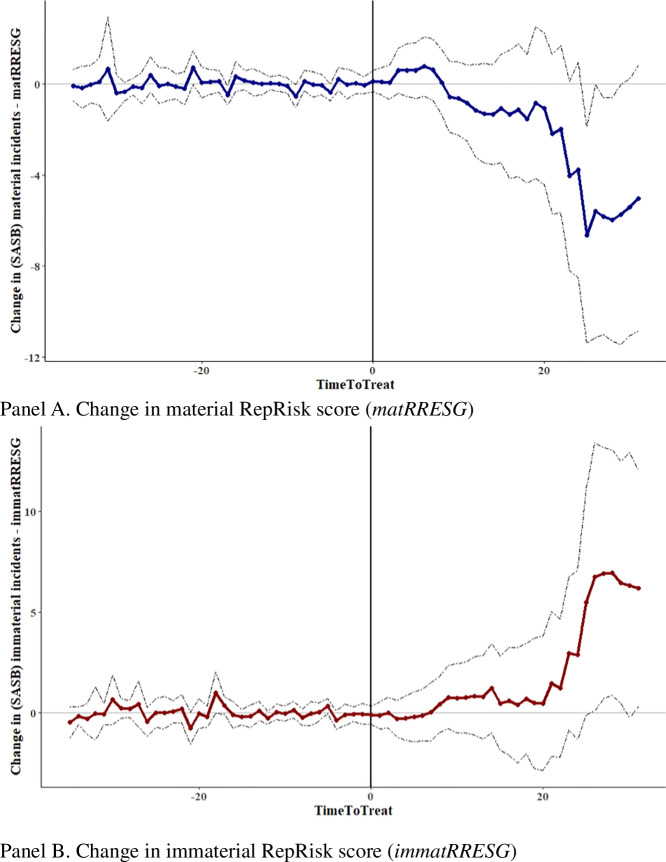
**Evolution of the** **ATT** **around the release of a SASB materiality standard** ($\boldsymbol e$ **=** **0**). The coefficients $\theta \left(e\right)$ are shown for *matRRESG* in blue and for *immatRRESG* in red. The dotted lines represent 95 percent confidence intervals

Figure 3 plots the cumulative *ATT* $\theta \left(e\right)$ for exposure lengths $e$ from −35 to + 31 months relative to a SASB standard release month ($e$ = 0). For instance, for the healthcare sector (*g* = healthcare), $e$ is zero for July 2013 and for the services sector $e$ is zero for December 2014. Panel A of Fig. 3 shows that the *ATT* of the SASB materiality classifications on *matRRESG* begins to evolve 10 to 12 months after the SASB standard release dates and then strengthens over time, leading to fewer, less severe, and less far-reaching material ESG incidents on average (as indicated by a decrease in *matRRESG*). By contrast, the *ATT* when using *immatRRESG* as the dependent variable in Panel B shows that, on average, the occurrence of immaterial incidents increases over time. Numerically speaking, *matRRESG* at + 30 months decreases by 6.0 points for material topics (see also column 1 in Panel B of Table 2) but increases by 6.9 points for immaterial topics (*immatRRESG*; see also column 2 in Panel B of Table 2). These findings support H1 and H2 and indicate that the SASB standards prompted a change in firms’ sustainability performance that prioritized material sustainability topics over immaterial ones.

Next, we provide evidence to support the parallel trends assumption, which is crucial in difference-in-differences designs. Panels A and B of Fig. 3 illustrate that the plotted coefficients of *ATT* $\theta \left(e\right)$ in the pre-release period differ insignificantly from zero. In other words, we find no pre-release differences in both material and immaterial sustainability performance between treated and untreated (or rather not-yet-treated) firms. This finding is important, as it mitigates concerns that certain industries were already more sensitive to sustainability topics (because of, for example, higher stakeholder pressure) before the SASB standard releases, and it suggests the inherently untestable parallel trends assumption.

Finally, we estimate the overall treatment effect ${\theta }^{Overall}$, which is an aggregated parameter (across the group and time dimensions) for the effect of SASB standard releases on subsequent material and immaterial sustainability performance. Panel A of Table 2 summarizes ${ATT \theta }^{Overall}$, and Panel B shows *ATT* $\theta \left(e\right)$ at different exposure lengths $e$. The results in Panel A again support H1 and H2 and show a negative and significant overall effect for *matRRESG* (−1.9221) and a positive and significant overall effect for *immatRRESG* (1.9187), which are both increasing with the length of exposure $e$. (See coefficients in Panel B at $e$ = + 25 and + 30, respectively.) Given the sample mean of 20.90 (Panel C of Table 1) for *matRRESG*, the decrease of 1.9221 points depicts an average decrease in the material incident score of 9.2 percent. For immaterial incidents, given the sample mean of 11.44 (Panel C of Table 1), the increase of 1.9187 points equates to a 16.8 percent average increase.

**Table 2:** Overall and dynamic treatment effect of SASB standard releases

	*matRRESG*	*immatRRESG*
	(1)	(2)
**Panel A**		
Overall treatment effect: ${\theta }^{Overall}$ for all $e$ > 0	−1.9221* (0.7028)	1.9187* (0.7626)
**Panel B**		
Dynamic treatment effect: $\theta \left(e\right)$ with $e$ = −35	−0.0662 (0.2240)	−0.4666 (0.2450)
Dynamic treatment effect: $\theta \left(e\right)$ with $e$ = −25	−0.0708 (0.2643)	0.0040 (0.2440)
Dynamic treatment effect: $\theta \left(e\right)$ with $e$ = −15	0.1603 (0.1319)	−0.2017 (0.1235)
Dynamic treatment effect: $\theta \left(e\right)$ with $e$ = −5	−0.3755 (0.1187)	0.3372 (0.1262)
Dynamic treatment effect: $\theta \left(e\right)$ with $e$ = + 5	0.5903 (0.3770)	−0.1756 (0.4095)
Dynamic treatment effect: $\theta \left(e\right)$ with $e$ = + 15	−1.0797 (0.8821)	0.4794 (0.7855)
Dynamic treatment effect: $\theta \left(e\right)$ with $e$ = + 25	−6.6350* (1.6144)	5.4928* (1.7824)
Dynamic treatment effect: $\theta \left(e\right)$ with $e$ = + 30	−5.4228 (1.8368)	6.3358* (2.0486)
Dynamic treatment effect: $\theta \left(e\right)$ with $e$ = + 31	−5.0331 (1.8409)	6.1993 (2.0798)
Control group	Not-yet treated	Not-yet treated
Estimator	Doubly robust	Doubly robust
Observations	24,408	24,408

This table reports results from estimating the staggered difference-in-differences model (Eq. (1)) with *matRRESG* and *immatRRESG* as the dependent variable. Following Callaway and Sant’Anna (2021), all effects are estimated doubly robust with firm-level clustered bootstrapped-based standard errors, which are provided in parentheses. * indicates statistical significance at least at the 5% level.

By conducting a leave-one-sector-out approach (Appendix D), we further mitigate concerns that our findings are driven by industry-specific regulations (e.g., mandatory mine-safety disclosures as examined by Christensen et al. (2017) or increased public scrutiny of sectors associated with sustainability issues that are perceived as sensitive, such as emissions or human rights). In sum, we find that SASB’s materiality classifications shifted managers’ focus from immaterial to material sustainability topics, prompting a trade-off between material and immaterial sustainability performance

### Investor and manager pre-SASB disagreement on materiality

In this section, we refine identification by examining potential heterogeneity in the treatment effect. Specifically, we argue that the SASB standards introduced a common understanding of materiality between investors and managers, guiding the latter in decision-making. In sectors where pre-SASB disagreement about materiality between investors and managers was higher, this effect was likely more pronounced, as the standards reduced managerial discretion in defining materiality and aligned firms’ sustainability priorities more closely with investor expectations. For this reason, we would expect that the observed reduction in *matRRESG* and increase in *immatRRESG* is particularly concentrated in firms operating in sectors showing higher pre-SASB disagreement between investors and managers.

To test for this effect, we accessed (upon our request to the SASB) the standard setter’s Industry Working Group data and calculated the disagreement between firm and investor representatives regarding the materiality of sustainability topics. For any given topic and industry, the working group data allows us to identify whether managers and investors agreed with the initial SASB materiality classifications derived in the internal research phase. For instance, in the e-commerce industry (part of the consumer goods sector), both manager and investor representatives agreed 100 percent that the topic “data security” is material. By contrast, in the healthcare delivery industry (part of the healthcare sector), 50 percent of firm representatives voted for “pricing and billing transparency” as a material topic, whereas 100 percent of investor representatives voted for it.

After aggregating disagreement at the sector level by averaging industry-level disagreement, we observe that the highest disagreement is in the healthcare sector, with an average disagreement of 30 percentage points and the lowest disagreement is in the financials and transportation sectors, with 14 percentage points on average. In the last step, we classify sectors with above-average disagreement (across all sectors) as high-disagreement sectors and firms with below-average disagreement as low-disagreement sectors.^10^ Accordingly, we classify firms in the healthcare, services, renewable resources, and infrastructure sectors as high-disagreement sectors and firms in the financials, technology and communications, extractives and minerals processing, transportation, resource transformation, consumer goods, and food and beverage as low-disagreement sectors. Panel A of Table 3 presents the average level of disagreement across sectors and the final classification into high- and low-disagreement sectors.

We use firm-year data between 2011 and 2020 to estimate the following model (firm and time subscripts omitted):

2 $$matRRESG\;\mathrm{or}\;immatRRESG=\beta_0+\beta_1Treated+\beta_2Treated\times HighDisagreement+\sum\beta_iX_i+\sum\beta_j{FixedEffects}_j+\varepsilon.$$

In Eq. (2), the outcome variables are *matRRESG* or *immatRRESG*. The coefficient of interest is ${\beta }_{2}$, where *Treated* is coded one starting in the year in which the corresponding SASB standard is released in the sector a firm operates in and zero otherwise. *HighDisagreement* is a binary indicator that is one for firms in high-disagreement sectors and zero for firms in low-disagreement sectors. The control vector ${X}_{i}$ contains time-varying controls at the firm level. Specifically, we control for firm size (*Total Assets*) because larger firms have a greater willingness and ability to implement sustainability policies. We include return on assets (*ROA*), as profitable firms can invest more in new sustainable technologies. Sales growth (*Sales Growth*) controls for sustainability impacts due to firm growth. We include institutional ownership (*Inst. Ownership*) because large investor groups can influence sustainability policies through engagement. Firm-fixed effects control for time-invariant factors within firms, and year-fixed effects control for time-specific shocks or trends that affect sustainability performance across all firms, for example, economic conditions. We estimate Eq. (2) using OLS regression with clustered heteroscedasticity-robust standard errors at the firm level.

**Table 3:** Pre-SASB disagreement effect

**Panel A**		
Sector	Average disagreement (%)	High-disagreement sector
Consumer Goods	17	No
Extracting and Minerals Processing	15	No
Food and Beverage	18	No
Financials	14	No
Healthcare	30	Yes
Infrastructure	23	Yes
Renewable Resources	25	Yes
Resource Transformation	19	No
Services	23	Yes
Technology	15	No
Transportation	14	No
Sample average	19.36	
**Panel B**		
	*matRRESG*	*immatRRESG*
	(1)	(2)
*Treated* × *HighDisagreement*	−4.307*** (0.964)	1.680** (0.787)
Controls	Yes	Yes
Firm-fixed effects	Yes	Yes
Time-fixed effects	Yes	Yes
Firm clustered standard errors	Yes	Yes
Observations	5,028	5,028
Adjusted R^2^	0.066	0.023
F Statistic	12.000***	8.730***

This table reports results from estimating the staggered difference-in-differences model (Eq. (2)) with *matRRESG* and *immatRRESG* as the dependent variable. *Treated* is a binary indicator, coded 1 starting in the year in which the corresponding SASB standard is released in the sector a firm is operating in and 0 otherwise. *HighDisagreement* = 1 for firms in high-disagreement sectors and 0 otherwise. High-disagreement sectors are healthcare, services, renewable resources, and infrastructure. Low-disagreement sectors are financials, technology, extracting and minerals processing, transportation, resource transformation, consumer goods, and food and beverage. Heteroscedasticity-robust standard errors clustered at the firm level are provided in parentheses. ***, **, and * indicate statistical significance at the 1%, 5%, and 10% levels, respectively

Panel B of Table 3 summarizes the estimated average effect of SASB standard releases on subsequent material and immaterial incident scores of high-disagreement sectors. Focusing on material incidents, column 1 shows that firms in high-disagreement sectors have a significantly lower *matRRESG* (−4.307) following the SASB standard release compared to firms in sectors with low pre-SASB disagreement on materiality classifications. These findings support H1 and indicate that SASB materiality classifications are most beneficial in sectors with high pre-SASB disagreement on the financial materiality of sustainability topics. We further test whether firms in high-disagreement sectors also show higher immaterial incidents after the SASB standard releases, which would indicate a decline in immaterial sustainability performance induced by resource allocation (H2). In column 2 of Table 3, we find a significantly higher *immatRRESG* for firms in high-disagreement sectors (+ 1.680) compared to those in low-disagreement sectors. This indicates a reallocation of resources from immaterial to material sustainability topics, thus supporting H2. In sum, the positive (negative) effect of SASB materiality classifications on (im)material sustainability incidents is concentrated in firms in high-disagreement sectors. This finding tightens identification, as the common understanding of materiality on sustainability topics—as reinforced by SASB standards—is most beneficial when the pre-SASB-release disparity between investors’ and firms’ understandings of materiality is more pronounced.

### Quasi-never-treated control group and alternative outcome variables

We now address concerns of measurement bias in the RepRisk data (Johnson 2020) by using two alternative outcome variables to measure sustainability performance. We first use Scope 1 (denoted *IntensityScope1*) and Scope 2 (denoted *IntensityScope2*) emission intensity data from ISS ESG. Second, we collect yearly workplace safety or health violation fines from Violation Tracker divided by sales (denoted *FineSafetyVio*) and use a dummy indicating whether a firm had at least one fine (denoted *FineSafetyVioDummy*).^11^ We observe emission data for 4,403 firms, and Violation Tracker data show that 2,445 fines were charged for workplace safety or health violations within our sample period of 2011 to 2020.

Leveraging the structure of the SASB standards, we construct a quasi-never-treated control group for each of the two topics.^12^ Specifically, we assign firms to either the treated group or our quasi-never-treated control group based on whether greenhouse gas emissions or workplace safety or health violations (i.e., SASB General Issue Categories (GIC): *GHG Emissions* and *Employee Health and Safety*) are considered material in their industry, according to SASB’s materiality classifications. Appendix E shows the corresponding industry lists. Accordingly, we construct two indicator variables: (i) *GHGIndustry*, which is one for firms in industries for which the topic *GHG Emissions* is material and zero for all remaining firms, and (ii) *WorkplaceSafetyIndustry*, which is one for firms in industries for which the topic *Employee Health & Safety* is material and zero for all remaining firms. Consistent with our previous results, we expect firms in treated industries (*GHGIndustry* or *WorkplaceSafetyIndustry*) to exhibit lower emission intensity and fewer safety violations, on average, compared to firms in our quasi-never-treated control groups. We test this expectation by estimating the following models (firm and time subscripts omitted):

3 $$IntensityScope1\text{ or }IntensityScope2={\beta }_{0}+{\beta }_{1}Treated+{\beta }_{2}Treated\times GHGIndustry+\sum {\beta }_{i}{X}_{i}+\sum {\beta }_{j}{FixedEffects}_{j}+\varepsilon ;$$

4 $$FineSafetyVio={\beta }_{0}+{\beta }_{1}Treated+{\beta }_{2}Treated\times WorkplaceSafetyIndustry+\sum {\beta }_{i}{X}_{i}+ \sum {\beta }_{j}{FixedEffects}_{j}+\varepsilon .$$

Compared to Eq. (2) we replace the outcome variable with *IntensityScope1* or *IntensityScope2* in Eq. (3) and with *FineSafetyVio* and *FineSafetyVioDummy* in Eq. (4). In addition, we replace *HighDisagreement* with *GHGIndustry* when using emission intensity and with *WorkplaceSafetyIndustry* when using workplace safety or health violation fines as outcome variables, respectively. All other specifications remain the same.

As shown in columns 1 and 2 of Table 4, the coefficient of interest, *Treated* × *GHGIndustry*, is negative and statistically significant when *IntensityScope1* and *IntensityScope2* are the outcome variables. This indicates that carbon emission intensity is lower following SASB standard releases in industries with GHG emissions deemed a material topic compared to industries where it is not. Regarding *FineSafetyVio*, the results in column 3 of Table 4 also support our main findings. More precisely, the coefficient for *Treated* × *WorkplaceSafetyIndustry* is significant at the 10 percent level, indicating that treated firms’ fine ratio is lower compared to quasi-never-treated control firms. In column 4, we use *FineSafetyVioDummy* as the dependent variable. The coefficient for *Treated* × *WorkplaceSafetyIndustry* is again negative and significant at the 5 percent level, which further supports our main findings.^13^ Taken together, these findings mitigate concerns about measurement bias in RepRisk scores as they provide similar insights when using alternative outcome variables.

**Table 4:** Alternative outcome variables

	*IntensityScope1*	*IntensityScope2*	*FineSafetyVio*	*FineSafetyVioDummy*
	(1)	(2)	(3)	(4)
*Treated* × *GHGIndustry*	−246.600*** (89.27)	−45.560** (20.36)		
*Treated* × *WorkplaceSafetyIndustry*			−2.940* (1.561)	−0.070** (0.032)
Controls	Yes	Yes	Yes	Yes
Firm-fixed effects	Yes	Yes	Yes	Yes
Time-fixed effects	Yes	Yes	Yes	Yes
Firm clustered std. errors	Yes	Yes	Yes	Yes
Observations	4,403	4,403	5,028	5,028
Adjusted R^2^	0.033	0.012	0.029	0.013
F Statistic	3.60***	3.38***	1.74**	3.79***

This table reports results from estimating the staggered difference-in-differences models with variations of *IntensityScope* (Eq. (3)) and *FineSafetyVio* (Eq. (4)) as the dependent variable. *Treated* is a binary indicator, coded 1 starting in the year in which the corresponding SASB standard is released in the sector a firm is operating in and 0 otherwise. *GHGIndustr*y and *WorkplaceSafetyIndustr*y are binary indicators set to one for firms operating in industries where the respective topics are material according to the SASB standards. (See Appendix D for more details.) Heteroscedasticity-robust standard errors clustered at the firm level are provided in parentheses. ***, **, and * indicate statistical significance at the 1%, 5%, and 10% levels, respectively

We address concerns that our results are driven by firms adopting and disclosing in accordance with SASB standards in Appendix F. We show that the changes in firms’ material and immaterial sustainability performance are not driven by firms that subsequently adopt the SASB standards for external reporting. This supports the notion that managers begin reallocating resources toward material sustainability topics as early as the release of the standards.

## Mechanism tests

Having identified the effect of SASB standard releases on material and immaterial sustainability performance, we now provide cross-sectional evidence that the observed changes in sustainability incidents are driven by firms’ internal resource allocation decisions. We also test whether the mechanisms discussed in the hypothesis development section (shareholder activism and sustainability-linked compensation) amplify those allocation decisions. Identifying correlations between our mechanism and outcome variables provides insights into the factors driving firms’ resource allocations and decision-making. For these additional analyses, we specify the following model (firm and time subscripts omitted):

5 $$Outcome={\beta }_{0}+\sum {\beta }_{m}TimeToTreatX\times {Mechanism }_{m}+\sum {\beta }_{i}{X}_{i}+\sum {\beta }_{j}{FixedEffects}_{j}+\varepsilon .$$

In Eq. (5), *Outcome* is the outcome variable as described in Sects. 6.1 to 6.3, respectively. *TimeToTreatX* is a categorical variable indicating the years since the SASB standard release in each sector. (See the release dates in Appendix A.) The baseline group is *TimeToTreat0*, which is the year of the respective SASB standard release. For example, *TimeToTreat1* is the first year after the respective SASB standard release. We include all observations up to *TimeToTreat4*, that is, four years after the respective SASB standard release. We then interact *TimeToTreatX* with a mechanism variable (denoted *Mechanism*, described in Sects. 6.1 to 6.3). The ${\beta }_{m}$ coefficients indicate whether and how the tested mechanism affects the outcome variable over time. ${X}_{i}$ includes the identical control variables as in Eq. (2). We apply firm-fixed and time-fixed effects and heteroscedasticity-robust standard errors clustered at the firm level.

### Internal sustainability policy changes

We first examine whether firms adjust their internal sustainability policies—used as proxies for resource allocation—following the release of SASB standards. We do so because firms’ internal changes of sustainability policies could explain the changes in sustainability incidents observed in Sect. 5. SASB guidance on the materiality of sustainability topics, combined with managers’ fiduciary duty to maximize firm value, supports a shift toward material sustainability policies and away from immaterial ones. We further argue that these internal policy changes are amplified for high-exposure firms because of heightened learning effects and higher potential pressure by shareholders (Christensen et al. 2017; Fiechter et al. 2022). Accordingly, we define high-exposure firms as those with material Refinitiv policy scores *below* the sector median or immaterial Refinitiv policy scores *above* the sector median in the year prior to the SASB standard release.

We construct Refinitiv policy scores by manually linking Refinitiv’s ESG policies to the respective SASB Materiality Map dimension within a GIC. (See Appendix G for a detailed description.) On a yearly basis, for each firm in a given sector, the identified material and immaterial policy scores are summed and then divided by the corresponding number of material and immaterial policies in the sector, respectively. Thus, we observe a material policy score (denoted *materialESGpol*) and an immaterial policy score (denoted *immaterialESGpol*) for each firm. Both variables replace the variable *Outcome* in Eq. (5). The *Mechanism* variable captures high-exposure firms and is denoted as *preSASBexp*. When examining material ESG policies, the indicator variable *preSASBexp* is one for firms with *materialESGpol below* the corresponding sector median of material policies in the year prior to the SASB release and zero otherwise. When examining immaterial ESG policies, the indicator variable *preSASBexp* is one for firms with *immaterialESGpol above* the corresponding sector median of immaterial policy scores.

Table 5 shows the results. After replacing *Outcome* with *materialESGpol* in Eq. (5), we find positive, significant, and economically meaningful coefficients for *TimeToTreatX* × *preSASBexp* starting in the first year after the SASB standard release (column 1). This suggests that high-exposure firms significantly allocate resources to material sustainability policies, aiming to improve material sustainability performance. In column 2, supporting our argument of resource reallocation, we find significant negative coefficients for *TimeToTreatX* × *preSASBexp* with *immaterialESGpol* as the outcome variable. We interpret these findings as follows. Immediately after the SASB standard releases, high-exposure firms begin reallocating resources from immaterial to material ESG policies. Consequently, material sustainability performance starts to improve while immaterial performance declines over time. In the long run, these adjustments in firms’ internal sustainability policies align with our main findings, showing fewer, less severe, and far-reaching material sustainability incidents (i.e., a reduction in *matRRESG*) but simultaneously more and increasingly severe and far-reaching immaterial ones (i.e., an increase in *immatRRESG*). Overall, our findings document that firms prioritize material sustainability policies over immaterial ones following the SASB standard releases, revealing a pattern that supports our main findings.

**Table 5:** Firms’ material and immaterial sustainability policies

	*materialESGpol*	*immaterialESGpol*
	(1)	(2)
*TimeToTreat1* × *preSASBexp*	2.428^**^ (1.037)	−3.796^***^ (1.259)
*TimeToTreat2* × *preSASBexp*	5.178^***^ (1.336)	−4.873^***^ (1.413)
*TimeToTreat3* × *preSASBexp*	7.129^***^ (1.576)	−6.861^***^ (1.701)
*TimeToTreat4* × *preSASBexp*	7.577^***^ (1.789)	−7.694^***^ (1.845)
Controls	Yes	Yes
Firm-fixed effects	Yes	Yes
Time-fixed effects	Yes	Yes
Firm clustered standard errors	Yes	Yes
Observations	2,297	2,297
Adjusted R^2^	0.067	0.075
F Statistic	5.040^***^	5.420^***^

This table reports results from estimating the staggered difference-in-differences model (Eq. (5)) with *materialESGpol* and *immaterialESGpol* as the dependent variable. In column 1, *preSASBexp* = 1 for firms that show a *below* sector median performance in material sustainability policies in the year prior to the SASB standard release and 0 otherwise. In column 2, *preSASBexp* = 1 for firms that show an *above* sector median performance in immaterial sustainability policies in the year prior to the SASB standard release and 0 otherwise. *TimeToTreat* is a categorical variable indicating the years passed since SASB standard releases. (See release dates in Appendix A.) Heteroscedasticity-robust standard errors clustered at the firm level are provided in parentheses. ***, **, and * indicate statistical significance at the 1%, 5%, and 10% levels, respectively

### Shareholder pressure

Our hypotheses are based on the argument that shareholder proposals raise managers’ awareness of sustainability topics (Cunat et al. 2012; Diaz-Rainey et al. 2024; Dimson et al. 2015; Flammer et al. 2021). Thus, we test whether shareholder activism affects firms’ subsequent sustainability performance. In light of the SASB materiality classifications, firms might want to focus more on material sustainability topics following shareholder activism because these topics, by definition, matter more to shareholders. Accordingly, more resources are moved away from immaterial sustainability topics. We expect the reduction (increase) in *matRRESG* (*immatRRESG*) to vary with the level of shareholder activism. We use the FactSet shareholder proposal dataset to construct a shareholder activism variable. Restating Eq. (5), we denote *preSASBprop* (replaces *Mechanism*) as equal to one when shareholders file at least one ESG proposal at the last annual general meeting prior to the respective SASB standard release and zero otherwise.

Table 6 summarizes the results. Column 1 (*matRRESG* replaces *Outcome* in Eq. (5)) shows that firms facing shareholder pressure at the annual general meeting before the SASB standard release reduce material incidents more than firms that do not face such pressure. This trend first becomes significant two years after the standard release and increases (both in magnitude and statistical significance) in the following years. By contrast, column 2 (*immatRRESG* replaces *Outcome* in Eq. (5)) indicates that firms facing shareholder pressure experience a greater increase in immaterial sustainability incidents in the long run. While this trend seems to develop after two years, it attains significance after four years. Since shareholder activism targets directors and CEOs, we argue that the resource reallocation from immaterial to material topics is driven by top management. BlackRock exemplifies this outcome, as CEO Larry Fink explicitly holds top management accountable for sustainability performance. He states in his shareholder letter that “a company’s ability to manage [sustainability] matters demonstrates the leadership and good governance that is so essential to sustainable growth” (Fink 2018). In sum, our findings support the view that shareholder pressure can explain our findings in Sect. 5 that firms focus on material sustainability topics after a SASB release.

**Table 6:** Shareholder pressure and changes in sustainability performance

	*matRRESG*	*immatRRESG*
	(1)	(2)
*TimeToTreat1* × *preSASBprop*	0.271 (1.133)	−1.037 (0.899)
*TimeToTreat2* × *preSASBprop*	−2.596^*^ (1.396)	0.864 (1.062)
*TimeToTreat3* × *preSASBprop*	−3.317^*^ (1.807)	1.068 (1.239)
*TimeToTreat4* × *preSASBprop*	−4.024^***^ (1.438)	2.213^**^ (0.991)
Controls	Yes	Yes
Firm-fixed effects	Yes	Yes
Time-fixed effects	Yes	Yes
Firm clustered standard errors	Yes	Yes
Observations	2,297	2,297
Adjusted R^2^	0.034	0.025
F Statistic	4.790^***^	3.360^***^

This table reports results from estimating the staggered difference-in-differences model (Eq. (5)) with *matRRESG* and *immatRRESG* as the dependent variable. For all post-SASB years, *preSASBprop* = 1 when shareholders filed at least one ESG proposal at the last annual general meeting before the respective SASB standard release and 0 otherwise. *TimeToTreat* is a categorical variable indicating the years passed since SASB standard releases. (See release dates in Appendix A.) Heteroscedasticity-robust standard errors clustered at the firm level are provided in parentheses. ***, **, and * indicate statistical significance at the 1%, 5%, and 10% levels, respectively

### Sustainability-linked compensation

Sustainability-linked compensation might also explain the divergence between material and immaterial sustainability performance. On the one hand, recent research suggests that sustainability-linked compensation improves overall sustainability performance, measured by relative improvements in ESG ratings (Cohen et al. 2023). On the other hand, others suggest a weaker link (Berrone and Gomez-Mejia 2009) or even raise concerns that linking executive pay to sustainability performance hurts stakeholders by prioritizing the interests of investors (Bebchuk and Tallarita 2023). Here, by separating overall sustainability performance into material and immaterial performance, we test whether the effect of sustainability compensation resides mostly in material sustainability performance, and whether firms’ non-investor stakeholders may bear the costs found by Bebchuk and Tallarita (2023) as an offsetting consequence.

In Eq. (5), we replace *Mechanism* with *CompInitiator*, which is one if Refinitiv indicates that a firm implemented sustainability-linked executive compensation for the first time after the respective SASB standard release and zero otherwise. The rationale for this variable lies in the notion that sustainability-linked compensation, implemented after the release of SASB standards versus before (or not at all), is likelier to align with the materiality classifications outlined by SASB, redirecting the focus of executives on material sustainability topics.^14^

Column 1 of Table 7 (*matRRESG* replaces *Outcome* in Eq. (5)) supports this idea by demonstrating a significant decrease in material sustainability incidents four years after the standard release for firms that initiate sustainability-linked compensation post SASB release. Once more, the lag in the decline of material incidents reflects a similar trajectory observed in our primary analyses. Firms first need to shift resources toward initiatives and policies targeting material topics, ultimately resulting in a decrease in material incidents in the long run. We find no significant indication, however, for a trajectory in the opposite direction for immaterial incidents (*immatRRESG* replaces *Outcome* in Eq. (5)) among our sample firms (column 2), which would indicate the potential costs of sustainability-linked compensation for stakeholders, as suggested by Bebchuk and Tallarita (2023).

**Table 7:** Sustainability-linked compensation and sustainability performance

	*matRRESG*	*immatRRESG*
	(1)	(2)
*TimeToTreat1* × *CompInitiator*	0.433 (0.737)	−1.268^*^ (0.712)
*TimeToTreat2* × *CompInitiator*	−1.286 (0.979)	−0.613 (0.944)
*TimeToTreat3* × *CompInitiator*	−1.871 (1.176)	0.068 (1.095)
*TimeToTreat4* × *CompInitiator*	−2.525^**^ (1.190)	0.209 (1.017)
Controls	Yes	Yes
Firm-fixed effects	Yes	Yes
Time-fixed effects	Yes	Yes
Firm clustered standard errors	Yes	Yes
Observations	2,297	2,297
Adjusted R^2^	0.035	0.024
F Statistic	4.550^***^	2.720^***^

This table reports results from estimating the staggered difference-in-differences model (Eq. (5)) with *matRRESG* and *immatRRESG* as the dependent variable. *CompInitiator* = 1 for years after a firm initiates a sustainability compensation plan after SASB standard release and 0 otherwise. *TimeToTreat* is a categorical variable indicating the years passed since SASB standard releases. (See release dates in Appendix A.) Heteroscedasticity-robust standard errors clustered at the firm level are provided in parentheses. ** and * indicate statistical significance at the 5% and 10% levels, respectively

As a further check, we limit our sample to S&P 100 firms for which we observe detailed proxy statement data in the sample of Bebchuk and Tallarita (2023).^15^ We replace *Mechanism* with *ESGCompS&P100*, which is one for firms identified by Bebchuk and Tallarita (2023) as having sustainability-linked executive compensation in place as of 2020 and zero otherwise. In Eq. (5), we replace *Outcome* with *matRRESG* and *immatRRESG*, respectively. While column 1 of Table 8 weakly indicates a reduction in *matRRESG*, similar to Table 7, it also shows a significant increase in *immatRRESG* in column 2, supporting the effect emphasized by Bebchuk and Tallarita (2023).

**Table 8:** S&P100 sustainability-linked compensation and sustainability performance

	*matRRESG*	*immatRRESG*
	(1)	(2)
*TimeToTreat1* × *ESGCompS&P100*	−0.468 (1.419)	−0.055 (1.231)
*TimeToTreat2* × *ESGCompS&P100*	−1.177 (1.941)	0.500 (1.714)
*TimeToTreat3* × *ESGCompS&P100*	−1.010 (1.944)	0.776 (1.670)
*TimeToTreat4* × *ESGCompS&P100*	−4.142^*^ (2.373)	4.675^**^ (1.838)
Controls	Yes	Yes
Firm-fixed effects	Yes	Yes
Time-fixed effects	Yes	Yes
Firm clustered standard errors	Yes	Yes
Observations	433	433
Adjusted R^2^	0.071	0.053
F Statistic	3.590^***^	1.880^**^

This table reports results from estimating the staggered difference-in-differences model (Eq. (5)) with *matRRESG* and *immatRRESG* as the dependent variable. *ESGCompS&P100* = 1 if an S&P 100 firm has a sustainability compensation plan in 2020 according to its proxy statements and 0 otherwise. *TimeToTreat* is a categorical variable indicating the years passed since SASB standard releases. (See release dates in Appendix A.) Heteroscedasticity-robust standard errors clustered at the firm level are provided in parentheses. ** and * indicate statistical significance at the 5% and 10% levels, respectively

In sum, the findings support the idea that sustainability-linked compensation induces managers to neglect immaterial sustainability topics, as investing in them might be perceived as a waste of resources. Like shareholder pressure, our findings suggest that the incentives of sustainability-linked compensation are also responsible for resource reallocation from immaterial to material sustainability topics initiated by the top management. By separating overall sustainability outcomes into their material and immaterial components, our findings build a bridge between Cohen et al. (2023) and Bebchuk and Tallarita (2023) and provide a deeper understanding of sustainability-linked compensation and its impacts on sustainability performance.

## Conclusion

We examine whether and how the materiality classifications by the SASB prompt changes in U.S. firms’ sustainability performance and find that, following their releases, material sustainability incidents decrease but immaterial ones increase. This trade-off is more pronounced in the presence of higher pre-SASB disagreements between investors and managers on the materiality of sustainability topics and independent of firms’ adoption of the SASB standards. Our finding survives multiple challenges to identification and a host of robustness tests. We also show that these real effects align with changes in firms’ internal sustainability policies, supporting the notion that managerial decision-making shifts toward material sustainability topics at the time of the standard releases. Moreover, we plausibly explain why these observed changes in (im)material sustainability incidents occur: because of pre-SASB release shareholder pressure and firms’ adoption of sustainability compensation plans that favor material over immaterial topics. As shareholder activism and compensation plans target top management, our tests also indicate that this group drives the reallocation of resources from immaterial to material sustainability topics. Taken together, when a standard setter issues standards that deem certain sustainability topics as more relevant (i.e., material) than others, we document that firms follow suit, subsequently improving their performance on material topics. A potential downside of this guidance is that firms simultaneously reduce their sustainability performance on topics deemed immaterial by the standard setter.

Our finding of this trade-off carries a key policy implication. Stakeholders whose interests do not align with a standard setter’s materiality classifications could suffer as a result of this apparent neglect. While studies highlight trade-offs between sustainability and financial performance (Christensen et al. 2017; Downar et al. 2021), we uncover trade-offs between material and immaterial sustainability performance. This is a novel insight, as our evidence is consistent with firms diverting scarce resources away from topics considered immaterial to those deemed material. As new sustainability standards develop, especially those that may favor one stakeholder group over another, greater awareness of this downside effect is critical.

This policy implication also aligns with recent shifts toward double or multistakeholder materiality in sustainability disclosure (Baumüller and Sopp 2021; CSRD 2022; Friedman and Ormazabal 2024), which is a broader concept than the SASB’s notion of financial materiality. Emerging approaches, like double materiality, may mitigate this downside effect by better balancing stakeholders’ diverse interests. Additionally, it is important to consider that investors’ preferences for material sustainability topics evolve. What is immaterial today can change, for instance, in response to cultural and social norms and legislation or a broader understanding of materiality. Therefore, topics regarded as immaterial by investors today could acquire financial significance in the future and become material.

## Figures and Tables

**Fig. 4:**
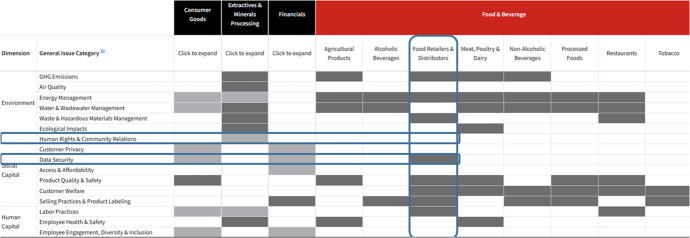
**SASB Materiality Map**. Source: https://www.sasb.org/standards/materiality-map/

**Fig. 5:**
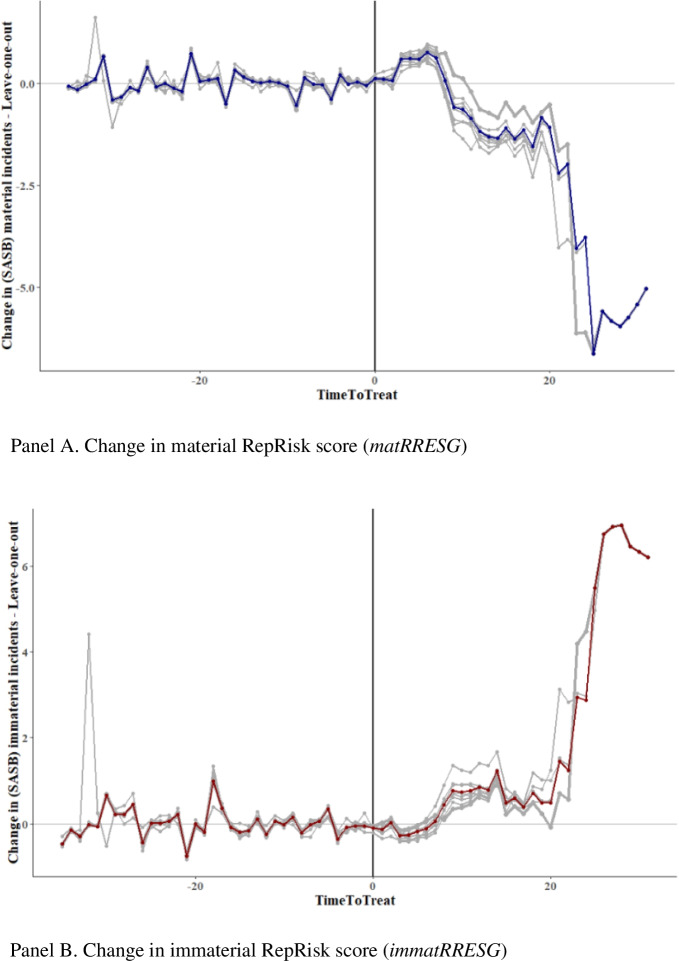
**Leave-one-sector-out approach**. The coefficients are shown for matRRESG in blue and for immatRRESG in red, respectively, and are the same as in Figure 3. The coefficients of the leave-one-sector-out approach are shown in gray

**Table 10:**
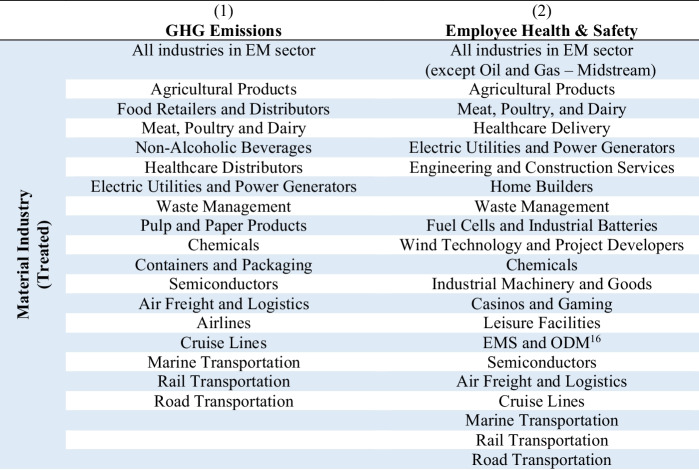
Treated groups

^a^Electronic Manufacturing Services and Original Design Manufacturing

**Table 11:**
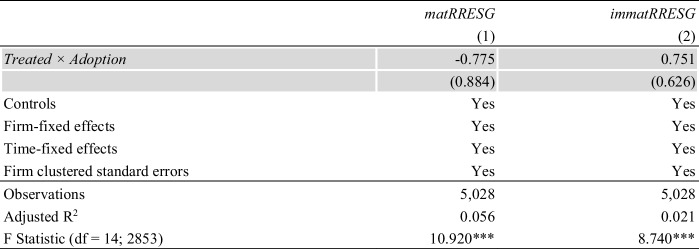
SASB standard adoption effect

This table reports results from estimating the staggered difference-in-differences model (Eq. (2); *HighDisagreement* is replaced with *Adoption*) with *matRRESG* and *immatRRESG* as the dependent variable. *Treated* is a binary indicator, coded 1 starting in the year in which the corresponding SASB standard is released in the sector a firm is operating in and 0 otherwise*. Adoption* = 1 for firms that have adopted the SASB standards in their reporting following the standard release and 0 otherwise. Heteroscedasticity-robust standard errors clustered at the firm level are provided in parentheses. ***, **, and * indicate statistical significance at the 1%, 5%, and 10% levels, respectively

**Table 12:**
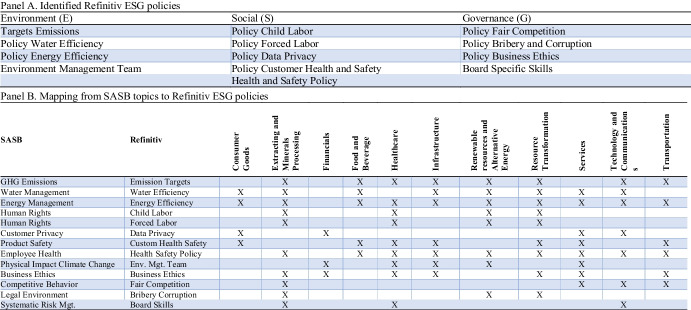
Construction of material and immaterial sustainability policies

## Data Availability

We attained all data used in this study from public sources, which are identified in the article.

## Appendix

Tables 9, 10, 11, 12.

Figures 4, 5.

### Appendix A. SASB standard release dates

Table 9 shows the sector release months. As can be seen, the first SASB sector (and associated industry standards, e.g., for the drug, retail, and managed care industries) release was in July 2013 for the healthcare sector. The last release was in March 2016 for the infrastructure sector.

**Table 9:** SASB standard release dates

Sector Name	Sector Release Month
Health Care	July 2013
Financials	February 2014
Technology and Communications	April 2014
Extractives and Minerals Processing	June 2014
Transportation	September 2014
Services	December 2014
Resource Transformation	March 2015
Consumer Goods	June 2015
Food and Beverage	September 2015
Renewable Resources and Alternative Energy	December 2015
Infrastructure	March 2016

### Appendix B. Variable description

**Table:**

Variable Name	Description	Source
**Identification variables**		
*Treated*	*Treated* = 1 for firms in all years beginning with and after the release of SASB standards for the respective sector in which the firm operates and 0 otherwise	Constructed SASB
*HighDisagreement*	*HighDisagreement* = 1 for firms in high-disagreement sectors (i.e., healthcare, services, renewable resources, and infrastructure) and 0 otherwise. The disagreement is calculated based on SASB Industry Working Group data. See Appendix A for release months and sector names	Constructed SASB
*Adoption*	*Adoption* = 1 for firms that adopted the standards in their reporting following the SASB standard release and 0 otherwise	Constructed SASB
*GHGIndustry*	*GHGIndustry* = 1 for firms in the GHG Emission column (see Appendix D) and 0 for all remaining firms	Constructed SASB
*WorkplaceSafetyIndustry*	*WorkplaceSafetyIndustry* = 1 for firms in the Employee Health and Safety column (see Appendix D) and 0 for all remaining firms	Constructed SASB
*TimeToTreatX*	$TimeToTreat$ is a categorical variable indicating the years *X* since a SASB standard release (see the release dates in Appendix A). The baseline group is *TimeToTreat*0, which is the year of the respective SASB standard release. *TimeToTreat*1 is the first year after the respective SASB standard release. We include all observations until *TimeToTreat*4, that is, four years after the respective SASB standard release	Constructed SASB
**Mechanism variables**		
p*reSASBexp*	*preSASBexp* = 1 for firms that show a *below* sector-median performance in material and an *above* sector-median performance in immaterial ESG policies prior to the SASB standard release, respectively and 0 otherwise	Refinitiv
*preSASBprop*	*preSASBprop* = 1 when shareholders filed at least one ESG proposal at the last annual general meeting prior to the SASB standard release and 0 otherwise	FactSet
*CompInitiator*	*CompInitiator* = 1 if a firm implements sustainability-linked executive compensation for the first time after the respective SASB standard release and 0 otherwise	Refinitiv
*ESGCompS&P100*	*ESGCompS&P100* = 1 if an S&P 100 firm has an ESG compensation plan in 2020 according to its proxy statements and 0 otherwise	Constructed following Bebchuk and Tallarita (2023)
**Outcome variables**		
*RRESG*	The *RRESG* includes the reach of information sources, the frequency, and timing of sustainability incidents as well as the risk incident content, i.e., severity, reach, and novelty of the ESG topics addressed. The *RRESG* ranges from 0 (lowest) to 100 (highest). A higher value indicates worse and far-reaching sustainability incidents are linked to a firm	RepRisk
*matRRESG*	*matRRESG* includes the reach of information sources, the frequency, and timing of material sustainability incidents as well as the material risk incident content, i.e., severity, reach, and novelty (newness) of the (SASB) material ESG topics addressed. The *matRRESG* ranges from 0 (lowest) to 100 (highest). A higher value indicates worse and far-reaching material sustainability incidents are linked to a firm	RepRisk
*immatRRESG*	*immatRRESG* is calculated by subtracting the *matRRESG* form the (overall) *RRESG*. Thus, a higher value indicates worse and far-reaching immaterial sustainability incidents are linked to a firm	Constructed
**Alternative outcome variables**		
*IntensityScope1*	*IntensityScope1* = Scope 1 GHG emissions divided by *Sales*	ISS ESG
*IntensityScope2*	*IntensityScope2* = Scope 2 GHG emissions divided by *Sales*	ISS ESG
*FineSafetyVio*	*FineSafetyVio* is the sum of yearly workplace safety or health violation fines a firm or one of its subsidiaries was charged with divided by *Sales*	Violation Tracker
**Sustainability policy variables**		
*materialESGpol*	*materialESPpol* matches Refinitiv E, S, and G policy scores with SASB GICs. All Refinitiv E, S, and G policy scores range between 0 and 100 (100 is the best performance). For any given firm, all policies that are categorized as material based on the SASB Materiality Map are summed and divided by the total number of material policies. As a result, *materialESGpol* is a score between 0 and 100. 100 is the best performance	Constructed
*immaterialESGpol*	*immaterialESGpol* matches Refinitiv E, S, and G policy scores with SASB GICs. All Refinitiv E, S, and G policy scores range between 0 and 100 (100 is the best performance). For any given firm, all policies that are categorized as immaterial based on the SASB Materiality Map are summed and divided by the total number of immaterial policies. As a result, *immatESGpol* is a score between 0 and 100. 100 is the best performance	Constructed
*Targets/Emissions*	Has the company set targets or objectives to be achieved on emission reduction? In scope are the short-term or long-term reduction target to be achieved on emissions to land, air, or water from business operations	Refinitiv
*Policy Water Efficiency*	Does the company have a policy to improve its water efficiency? In scope are the various forms of processes, mechanisms, or procedures to improve water use in operation efficiently System or a set of formal documented processes for efficient use of water and driving continuous improvement	Refinitiv
*Policy Energy Efficiency*	Does the company have a policy to improve its energy efficiency? In scope are the various forms of processes, mechanisms, or procedures to improve energy use in operation efficiently System or a set of formal documented processes for efficient use of energy and driving continuous improvement	Refinitiv
*Environment Management Team*	Does the company have an environmental management team? In scope are any teams that perform the functions dedicated to environmental topics An individual or team at any level composed of employees, even if the name of the team is different performing implementation of the environmental strategy It is important to understand that the members of the team include employees of the company, who are operational on a day to day basis and are not the board committees (directors)	Refinitiv
*Policy Child Labor*	Does the company have a policy to avoid the use of child labor? Actions, programs, or initiatives to avoid child labor or the employment of children under legal working age for the company or its suppliers Consider information from industry code such as the Electronic Industry Citizenship Coalition code of conduct and Pharmaceutical Industry Principles Legal compliance data is considered	Refinitiv
*Policy Forced Labor*	Does the company have a policy to avoid the use of forced labor? Actions, programs, or initiatives to avoid forced or compulsory labor for the company or its suppliers Practices to avoid any work for which people are forced to do against their will Consider information from industry codes such as the Electronic Industry Citizenship Coalition code of conduct and Pharmaceutical Industry Principles Legal compliance data is considered	Refinitiv
*Policy Data Privacy*	Does the company have a policy to protect customer and general public privacy and integrity? Processes or initiatives in place by which it strives to respect the privacy of the general public and its customers in particular Includes safeguarding or securing the customer’s confidential data, such as account number, passwords, personal details, personal identification number, and any other sensitive information	Refinitiv
*Policy Customer Health & Safety*	Does the company have a policy to protect customer health and safety? Processes or initiatives in place by which it strives to market products which are fostering benefits to the consumer’s health and safety rather than putting it at risk Includes only product-related initiatives and not services Customer security is considered for media and telecommunication companies	Refinitiv
*Health & Safety Policy*	Does the company have a policy to improve employee health and safety within the company and its supply chain?	Refinitiv
*Policy Fair Competition*	Does the company describe in the code of conduct that it strives to be a fair competitor? Includes respecting other companies’ patents, copyrights, or intellectual properties, or avoiding anti-competitive behavior, price fixing, or other monopolistic tactics Information from the code of conduct section in any report	Refinitiv
*Policy Bribery and Corruption*	Does the company describe in the code of conduct that it strives to avoid bribery and corruption in all its operations? Policy in the code of conduct against bribery and corruption in its operations Consider information from the code of conduct section in any report Legal compliance data is not considered Includes inappropriate/improper payment, special favors, extortion, or kickback	Refinitiv
*Policy Business Ethics*	Does the company describe in the code of conduct that it strives to maintain the highest level of general business ethics? Information on respecting general business ethics or integrity Information from the code of conduct section	Refinitiv
*Board Specific Skills*	Percentage of board members who have either an industry-specific background or a strong financial background	Refinitiv
*Policy Fair Competition*	Does the company describe in the code of conduct that it strives to be a fair competitor? Includes respecting other companies’ patents, copyrights, or intellectual properties or avoiding anti-competitive behavior, price fixing, or other monopolistic tactics Information from the code of conduct section in any report	Refinitiv
**Covariates**		
*Total Assets*	*TotalAssets* Is the sum of cash and short-term investments; total receivables, net; total inventory; prepaid expenses and other current assets, total	Refinitiv
*ROA*	*ROA* measures a firm’s operating efficiency, regardless of its financial structure (in particular, without regard to the degree of leverage a firm uses), and is calculated by dividing a firm’s net income prior to financing costs by total assets	Refinitiv
*Sales Growth*	*Sales Growth* is a growth ratio. *Sales* in *t* = 0 is divided by *Sales* in *t* − 1	Refinitiv
*Inst. Ownership*	*Institutional Ownership* is the percentage of the primary shares issues by taking the latest ownership records reported in the last two years, summing them, and then dividing the sum by the total primary shares outstanding	Refinitiv

### Appendix C. SASB Materiality Map

Figure 4 displays an excerpt of the SASB Materiality Map. In the first three sector columns (consumer goods to financials), the dark gray fields depict highly material GICs on the sector level (e.g., the GIC *GHG Emissions* is highly material in the extractives and minerals processing sector). Light gray fields are material in the respective sector (e.g., the GIC *Access and Affordability* is material in the financial sector), while white fields show that a GIC is not material in the specific sector. As can be seen for the food and beverage sector (red box), each sector column can be expanded to the industry level, where dark gray fields indicate that a GIC is material and white fields indicate immaterial topics.

### Appendix D. Industry-specific regulations as threats to identification

Our main findings show that SASB’s materiality classifications shifted managers’ focus from immaterial to material sustainability topics and caused a trade-off between material and immaterial sustainability performance (see Sect. 5.1). However, industry-specific regulations (e.g., mandatory mine-safety disclosures as examined by Christensen et al. (2017)) or increased public scrutiny of sectors associated with sustainability issues that are perceived as sensitive (e.g., emissions or human rights) might bias our findings. Thus, we conduct a leave-one-sector-out approach and rerun the Callaway and Sant’Anna (2021) model, excluding all firms belonging to a specific sector per iteration, except for the first sector, healthcare, and the last, infrastructure (to keep the sample period constant). Figure 5 Panel A plots the coefficients for $\theta \left(e\right)$ observed in the previous section for *matRRESG* (blue in Panel A) and *immatRRESG* (red in Panel B). The $\theta \left(e\right)$ for each leave-one-out iteration are plotted in gray. As shown in Panels A and B of Fig. 5, over all iterations, leaving one sector out leads to insights similar to the main findings. This mitigates concerns that our findings are driven by increased public attention to certain sectors or industry-specific reporting mandates.

### Appendix E. Quasi-never-treated counterfactual construction

Table 10 shows the construction of the treated and quasi-never-treated control groups for the analysis in Sect. 5.3. Treated units require that a GIC (i.e., *GHG Emissions* or *Employee Health & Safety*) is material in their industry (see columns 1 and 2 below, respectively). Quasi-never-treated control groups consist of firms operating in industries for which the respective GIC (*GHG Emissions* or *Employee or Health & Safety*) is not classified as material. For *GHG Emissions*, we construct the binary indicator variable *GHGIndustry*, which is one for treated firms operating in industries shown in column 1 below and zero for firms operating in the remaining industries. For *Employee Health & Safety*, we construct the binary indicator variable *WorkplaceSafetyIndustry*, which is one for the treated firms operating in one of the industries shown column 2 below and zero for firms operating in the remaining industries.

### Appendix F. Adoption of SASB standards for external reporting

Our research question is whether the SASB standard releases affect firms’ material and immaterial sustainability performance. Our findings so far provide evidence that, on average, SASB guidance improves material sustainability performance (i.e., reduces *matRRESG*) while simultaneously lowering immaterial sustainability performance (i.e., increasing *immatRRESG*). One of our key arguments is that managers start using the information provided by SASB’s materiality classification *at the time of the standard release*, even if they have not yet committed to adopting the SASB standards for future external reporting. However, if the effect is concentrated among firms that subsequently decide to adopt SASB standards in their reporting, this would raise concerns that the effect arises *at the time of disclosure* rather than through SASB materiality classifications *at the time of the standard releases*.

There is evidence that internal usage of SASB’s materiality classifications can be independent of the later adoption of SASB standards for external reporting. First, from a theoretical perspective, if the costs of adopting SASB standards do not exceed the expected benefits (Verrecchia 1983), managers would refrain from disclosing SASB material information in external reports, even if they use them internally. Second, managers tend to avoid disclosing unfavorable information in their financial statements in general (Kothari et al. 2009a, b; Kothari et al. 2009a, b). Even when there are laws to report material climate topics (e.g., SEC Form 10-K), managers may still omit or obfuscate material sustainability information if it harms the firm or manager (Matsumura et al. 2022). Third, according to the SASB, the number of firms using SASB reporting metrics was low in the first years after SASB standard releases (SASB 2018). Therefore, if we find market-wide changes in material (or immaterial) sustainability performance, it is unlikely that these changes are driven only by a small portion of later-adopting firms.

In addition to these theoretical arguments, we can test for differences in our findings between SASB-adopting and non-adopting firms. To do so, we replace *HighDisagreement* in Eq. (2) with *Adoption*, a dummy variable that is one for firms that adopted the standards in their reporting until 2020 and zero otherwise. Table 11 shows that *Treated* × *Adoption* does not affect *matRRESG* or *immatRRESG* and thus suggests that our main findings are not concentrated among SASB-adopting firms. Because the decision to adopt the SASB standards is endogenous, we cannot argue for causal insights. However, besides our theoretical arguments supporting a market-wide effect of the SASB standard release, the results support our previous findings. In other words, it is not firm-specific disclosure following the adoption of SASB standards that drives our main findings but rather the SASB’s materiality classifications *at the time of the standard releases*.

### Appendix G. Sustainability policy changes in response to SASB releases

To test firms’ internal reactions to the SASB release, we use the Refinitiv ESG score framework and identify firms’ policies in the E, S, and G pillars. As can be seen in Panel A of Table 12, we map them to the respective SASB GIC categories (e.g., *GHG Emissions*). Next, we categorize sustainability policies as either material or immaterial based on the SASB Materiality Map and apply the generated average material and immaterial sustainability policies scores (as depicted in Panel B below) as outcome variables in Sect. 6.1. Summary statistics of the variables *materialESGpol* and *immaterialESGpol* can be found in Panel D of Table 1.

## Footnotes

1 For details, see https://sasb.org/implementation-primer/.

2 SASB standards follow the SEC’s definition of materiality (SASB, 2018).

3 Working group participants were listed in the inaugural documentation published by SASB as part of the standards’ development process (for the healthcare sector, this document is dated November 11, 2012).

4 Appendix C provides an excerpt from the SASB Materiality Map and depicts the materiality structure at the sector and industry levels.

5 Investors can use the SEC’s or other sustainability disclosure standards’ definition of materiality, gather relevant information through various channels (e.g., sustainability reports, SEC filings, or a firm’s homepage), and evaluate the collected information to define their own understanding of materiality.

6 We gratefully acknowledge the thoughtful feedback of an anonymous reviewer, who highlights that these trade-offs differ from those between financial and sustainability performance as discussed in the literature (e.g., Haffar and Searcy 2017). Additionally, we acknowledge that our subsequent (theoretical) arguments hold only under the assumption that material and immaterial sustainability topics are substitutes.

7 Consultations with RepRisk confirm that each incident is assessed and evaluated equally within RepRisk’s methodology, regardless of whether the SASB classifies it as material or immaterial. In other words, each tracked incident is initially evaluated based on the same parameters (i.e., severity, reach, and novelty) and only in the second step the incident is classified as material or immaterial, depending on the sustainability topic identified and the industry in which the firm operates.

8 We thank RepRisk for providing us with the data and code to calculate material and immaterial scores.

9 A related concern arises because other (albeit only a few) studies have used SASB standard releases in a staggered introduction setting. This introduces the issue of multiple hypotheses testing (Heath et al. 2023). To address this, we compare the observed *t*-statistics from our main analysis in Sect. 5.1 (*t* = 2.734 and *t* = 2.516 in Table 2 columns 1 and 2, respectively) with the adjusted *t*-statistics for staggered introduction designs provided by Heath et al. (2023). As a result, our findings are robust to previous tests with eight and four different outcome variables, respectively. Even though these are not high numbers, given the few studies on SASB standard releases using a staggered event design, these findings further mitigate concerns that our main findings occur by chance.

10 Our findings are robust when using the median or using only the four sectors with the highest and the four sectors with the lowest disagreement, respectively.

11 We focus on workplace safety or health violation fines exclusively for two reasons. First, they are by far the most frequent offense group (number of offenses exceeds 1,500) compared to the second most frequent offense group, environmental violations, with roughly 750 offenses, and with even less offenses for further groups. Second, of the three offense groups with most offenses reported, only workplace and health violations are linked directly to one SASB General Issue Category (GIC). For example, the group environmental violations reflects a variety of issues (e.g., greenhouse gas emissions or waste management) associated with many SASB GICs as shown in Appendix C. Thus, we cannot assign clear treatment and control indicators for this group.

12 A quasi-never-treated control group consists of firms operating in industries for which the respective GIC (i.e., *GHG Emissions* or *Employee or Health & Safety*) is not classified as material. Given that shareholder pressure on material topics increases managers’ focus on these topics, firms operating in industries in which *GHG Emissions* or *Employee or Health & Safety* are immaterial topics should be not pressured or incentivized to improve performance on these topics, thus serving as the control group.

13 In an untabulated test, we exclude firms from the metals and mining industry due to a specific safety record disclosure regulation (as examined by Christensen et al. (2017). The results stay qualitatively the same and support our previous results.

14 As such, our focus is on the adoption of a sustainability-linked compensation plan to explain future sustainability incidents and not the factors or conditions under which a firm decides to adopt such a plan in the first place.

15 For example, Bebchuk and Tallarita (2023, p. 48) specifically single out ESG compensation plans that “focus on narrow dimensions of a subset of relevant stakeholders … and disincentivize corporate leaders from focusing on many other aspects of stakeholder welfare.” However, the increase in topic-level detail comes at the expense of loss of timing-level detail, as Bebchuk and Tallarita (2023) only provide proxy statement data for 2020.
